# Long-term structural brain changes in adult rats after mild ischaemic stroke

**DOI:** 10.1093/braincomms/fcac185

**Published:** 2022-07-22

**Authors:** Warda Syeda, Charlotte M Ermine, Mohamed Salah Khilf, David Wright, Vanessa H Brait, Jess Nithianantharajah, Scott Kolbe, Leigh A Johnston, Lachlan H Thompson, Amy Brodtmann

**Affiliations:** The Florey Institute of Neuroscience and Mental Health, Parkville, Victoria, Australia; Melbourne Neuropsychiatry Centre, The University of Melbourne, Parkville, Victoria, Australia; The Florey Institute of Neuroscience and Mental Health, Parkville, Victoria, Australia; The Florey Institute of Neuroscience and Mental Health, Parkville, Victoria, Australia; Department of Neuroscience, Monash University, Clayton, Australia; The Florey Institute of Neuroscience and Mental Health, Parkville, Victoria, Australia; The Florey Institute of Neuroscience and Mental Health, Parkville, Victoria, Australia; Department of Neuroscience, Monash University, Clayton, Australia; The Melbourne Brain Centre Imaging Unit, The University of Melbourne, Parkville, Victoria, Australia; Department of Biomedical Engineering, The University of Melbourne, Parkville, Victoria, Australia; The Florey Institute of Neuroscience and Mental Health, Parkville, Victoria, Australia; The Florey Institute of Neuroscience and Mental Health, Parkville, Victoria, Australia

**Keywords:** rodent stroke, volumetric MRI, DTI, biomarkers, tensor-based morphometry

## Abstract

Preclinical studies of remote degeneration have largely focused on brain changes over the first few days or weeks after stroke. Accumulating evidence suggests that neurodegeneration occurs in other brain regions remote to the site of infarction for months and even years following ischaemic stroke. Brain atrophy appears to be driven by both axonal degeneration and widespread brain inflammation. The evolution and duration of these changes are increasingly being described in human studies, using advanced brain imaging techniques. Here, we sought to investigate long-term structural brain changes in a model of mild focal ischaemic stroke following injection of endothlin-1 in adult Long–Evans rats (*n* = 14) compared with sham animals (*n* = 10), over a clinically relevant time-frame of 48 weeks. Serial structural and diffusion-weighted MRI data were used to assess dynamic volume and white matter trajectories. We observed dynamic regional brain volume changes over the 48 weeks, reflecting both normal changes with age in sham animals and neurodegeneration in regions connected to the infarct following ischaemia. Ipsilesional cortical volume loss peaked at 24 weeks but was less prominent at 36 and 48 weeks. We found significantly reduced fractional anisotropy in both ipsi- and contralesional motor cortex and cingulum bundle regions of infarcted rats (*P* < 0.05) from 4 to 36 weeks, suggesting ongoing white matter degeneration in tracts connected to but distant from the stroke. We conclude that there is evidence of significant cortical atrophy and white matter degeneration up to 48 weeks following infarct, consistent with enduring, pervasive stroke-related degeneration.

## Teaser

Preclinical studies of remote degeneration post-stroke have largely focused on brain changes over the first few days or weeks following infarct. Syeda *et al.* present evidence of significant cortical atrophy and white matter degeneration using MRI in rats up to 48 weeks following mild cortical infarction, consistent with enduring, pervasive stroke-related degeneration.

## Graphical Abstract

**Graphical Abstract:**
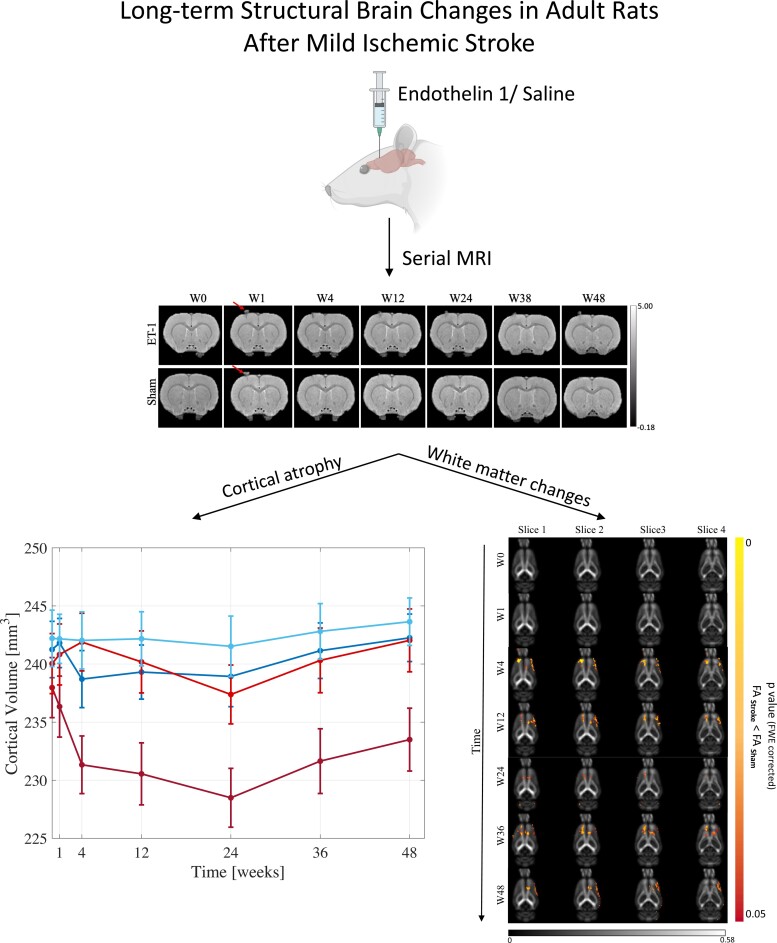

## Introduction

Ischaemic stroke is one of the leading causes of death and disability in the world. Acute ischaemic stroke is characterized by interrupted blood supply causing neuronal death (infarction) in the region of the occluded vascular territory. Brain infarction triggers a vigorous inflammatory response, accompanied by large-scale changes in brain network plasticity to facilitate functional recovery, associated with an eventual amelioration of microglial activation and restitution of neuronal activity to peri-infarct regions. However, it has been recognized for some time that secondary neurodegeneration (SND) may also eventuate, characterized by the progressive regional changes to areas functionally connected to but remote from the injury site.^1–3^ This latter phenomenon likely contributes to post-stroke brain atrophy and may contribute to post-stroke cognitive impairment and vascular dementia.^3–5^ Structural disconnections remote from the ischaemic lesions have been observed in the human brain including impaired connectivity of the cortex,^6^ hippocampus^7–9^ and thalamus.^10,11^ We recently followed up post-ischaemic stroke patients for 1 year and reported extensive white matter (WM) degeneration early after stroke^12^ as well as accelerated atrophy of the thalami, hippocampi and total brain volume.^4,5,13^

Animal studies of stroke increase our understanding of biological mechanisms underlying pathogenesis and progression of SND following an ischaemic event. Previous rodent studies have predominately used the middle cerebral artery occlusion (MCAo) model of ischaemic stroke. Unlike in humans, where the hippocampi and thalamic nuclei are largely supplied by the posterior cerebral artery (PCA), MCAo in rodents often results in large brain infarcts including involvement of the hippocampus,^9,14^ and thalamus,^15,16^ which are key cognitive hubs in human cognition. Thalamic and hippocampal atrophy have been reported in post-mortem longitudinal histological analyses up to 24 weeks following an ischaemic event^15,17^ and our group have reported hippocampal and especially thalamic atrophy continuing out to a year post-stroke.^4^ Rodent MRI studies have further improved our understanding of longitudinal brain changes post-stroke, for example, by showing changes in WM tracts in the ipsilateral hemisphere, in the cortex, external capsule and corpus callosum.^18–20^

However, the evolution of brain changes following a minor cortical or subclinical stroke remains poorly understood. This is significant because these subclinical strokes are strongly associated with cognitive decline.^21^ Subclinical or ‘silent’ brain infarction is currently under-represented in the animal literature. While minor stroke or transient ischaemic attack (TIA) may have only transient functional deficits, it is now known that even minor ischaemia induces long-term brain changes, including permanent microvascular tissue damage^22,23^ and high risk of early recurrence compared with the rest of the population^24,25^ (reviewed in Simmatis et al. 2019). Recent human studies showed that TIA patients had a greater rate of brain atrophy compared with healthy controls,^26^ with a third of TIA patients showing vascular cognitive impairment as early as 3 months following the insult.^27^ Our group recently used histological approaches to report prolonged cortical atrophy as well as prolonged microglial activation in areas remote to the injury in a rodent ischaemic model of minor stroke.^28^ However, longitudinal evolution of structural and WM changes in minor stroke remains unknown.

We sought to investigate the long-term structural brain changes in rats following small, cortical, endothelin-1 (ET-1)-induced infarcts in the right motor cortex of adult rats. We followed these animals with serial MRI over a clinically relevant period of 48 weeks. T_2_*-weighted (T_2_*w) images were used to map volumetric trajectories in cortex, hippocampus and thalamus. Additionally, post-stroke whole-brain WM changes were assessed using diffusion-weighted imaging (DWI). We hypothesized that bi-hemispheric, progressive neurodegeneration would be found in areas connected to the infarcted motor cortex and altered WM microarchitecture.

## Materials and methods

### Animals

Adult 22-week-old male Long–Evans rats were used in this study. The experimental design and procedures were approved by the Florey Institute for Neuroscience and Mental Health. All animals were housed under a 12 h light/dark cycle with ad libitum access to food and water and a period of acclimatization of 2 weeks was observed upon arrival to the animal facility. This work followed the recommendations of the Stroke Therapy Academic Industry Roundtable (STAIR) and the ARRIVE guidelines.^29^

### Surgical procedure: focal ischaemic stroke

Focal ischaemia was induced by ET-1 injection to the motor cortex of rats at 22 weeks of age, with the animals randomly allocated to one treatment group (i.e. ET-1 or sham injection). A detailed description of the surgical procedures can be found elsewhere.^28,30^ Briefly, prior to surgery, animals were anaesthetized with isoflurane (5% at 1 L/min) and placed in a stereotaxic frame (Kopf, Germany) where deep anaesthesia was maintained for the duration of the surgery (2% at 1 L/min). The ET-1 toxin (800 pmol, Auspep) for the lesioned animals (*n* = 14) or saline for the controls (*n* = 10) was injected into the motor cortex at two rostro-caudal locations: 0.5 and 2.0 mm rostral and 2.8 mm lateral to bregma and 1.5 mm below the surface of the brain. The sample size was set based on our previous experience with the ET-1 model.^28,31^ One sham rat was sacrificed during the study due to the presence of a tumour growth and was excluded from the analysis. All rats were sacrificed 48 weeks post-procedure after last *in vivo* MRI.

### MRI experiments

To acquire MRI data, rats were anaesthetized with isoflurane and placed in a rat cradle with tooth and ear-bars to fix head position. During scanning, rats were kept anaesthetized with a mixture of 1–2% isoflurane and oxygen. A small air balloon attached to a pressure transducer was placed under the chest to monitor respiration. Body temperature was continuously observed using a rectal probe and kept at 37°C via a hot water circulation system. MRI was performed *in vivo* at baseline (0 W), 1 week, 4 weeks, 12 weeks, 24 weeks, 36 weeks and 48 weeks post-stroke. At the 24-week timepoint, incomplete scans due to technical issues for three animals (1 sham/2 ET-1) were excluded from the study.

MRI was performed using a 4.7 T MRI with Avance III console and rat surface coil (Bruker, USA). Multiecho-T_2_*-weighted images were acquired using a 3D-MGE sequence^32^ with parameters: first echo time = 4 ms, echo-spacing = 4 ms, 20 echoes, repetition time, TR = 110 ms, matrix size = 176 × 128 × 70, and 150 µm isotropic resolution. Single-shell DWI was performed using a 2D DTI-EPI sequence with parameters: effective echo time, TE = 26 ms, repetition time, TR = 3000 ms, matrix size = 96 × 80, 28 slices, resolution = 350  µm× 350 µm × 350 µm, gradient duration, *δ*  = 5 ms, gradient separation, Δ  = 12 ms, *b* = 2500 s/mm^2^, 81 directions and four non-diffusion-weighted volumes.

### MRI analyses

#### Volumetric assessment

For volumetric analyses, multiecho-T_2_*w images were bias-field corrected^33^ and averaged across echoes to increase contrast. A registration-based approach was employed to segment regions-of-interests (ROIs) using the ANTs software package.^34^ Specifically, separate templates were constructed from averaged images at each timepoint per group.^35^ These templates were combined to create an unbiased study template. The study template was nonlinearly registered to the Waxholm-space rat brain atlas^36^ using SyN diffeomorphisms to segment brain, cortex, hippocampus and thalamus. Subject-to-template nonlinear registration was performed to delineate ROIs in the subject images. Manual corrections were performed if needed. Region volume was computed by voxel count.

#### Tensor-based morphometry

To assess stroke-induced structural brain changes, whole-brain voxel-wise tensor-based morphometry (TBM) analyses were performed at each timepoint separately.^37^ TBM is used to investigate localized volumetric changes at a voxel level by using the information in the log-Jacobian metric computed from SyN diffeomorphisms in the subject-to-template direction. The log-Jacobian metric quantifies volume expansion or shrinkage in the individual brain with respect to a reference template. A minimum deformation template (MDT) was constructed from the baseline template from stroke animals and seven sham templates to create a reference space for the registration. Each image was registered to the MDT and voxel-wise log-Jacobian maps were calculated to identify region-wise expansion or contraction as the brain warps from subject to MDT space.

#### Diffusion imaging

DWI images were skull-stripped, bias-field corrected and denoized in MRtrix.^38^ Diffusion tensor images (DTIs) were estimated to construct a study-specific DTI-template using the DTI-TK software.^39^ Diffusion parameter maps (FA: fractional anisotropy, MD: mean diffusivity, AD: axial diffusivity, RD: radial diffusivity) were calculated and warped to the DTI-template space. FA is a measure of anisotropic diffusion in WM bundles and ranges from 0 (isotropic diffusion) to 1 (highly anisotropic diffusion in ordered fibre bundles such as corpus callosum). MD describes the average diffusivity of water in tissue and AD and RD describe water diffusivity along and perpendicular to the principal direction of WM fibre bundles, respectively. For each animal, voxel-wise fibre orientation distributions (FODs) were computed and warped to the DTI-template space to generate an FOD-template. A tractogram seeded at ipsilesional motor cortex was generated from template FODs to estimate connectivity to other brain regions using the MRtrix software.

### Statistical analyses

#### Volumetric assessment

To determine effects of stroke on regional volumes over time, we implemented a region-wise linear mixed effects model in RStudio,^40^ with region volume as the dependent variable. Baseline total brain volume and total brain volume at the timepoint of interest were used as covariates. Group, side (ipsi/contralesional), timepoint (categorical variables) were the fixed effect. To address between-subject heterogeneity, random intercept and random slope (side) by subject were included in the model as random effects. All fixed effects interactions were included. For all tests, *P* < 0.05 was considered significant unless otherwise stated.

Follow-up cross-sectional analyses were separately performed at each timepoint. For each animal, regional volumes at each timepoint were normalized with respect to the baseline volume to account for the effects of variations in the baseline volumes. The between-group volume differences were assessed cross-sectionally using the normalized percentage volume estimates. We performed multiway ANOVA in MATLAB^41^ with group (stroke/sham) as between-subject, and lesion side (ipsi/contralesional) as within-subject factor. All two-factor interactions were included in the model. *Post hoc* pairwise comparisons were performed using MATLAB’s ‘*multcompare*’ function with HSD-correction.

#### TBM and diffusion parameters

To assess the long-term effects of stroke on brain structure, voxel-wise unpaired *t*-tests were performed for the log-Jacobian maps and each diffusion metric and timepoint using the FSL ‘randomize’ function with 5000 permutations, corrected for multiple comparisons and threshold-free cluster enhancement.^42^ All voxels with *P* < 0.05 after correction were considered significant.

### Data availability

All data are available upon request.

## Results

### ET-1 model of focal ischaemic stroke and cortical atrophy post-stroke

**Figure 1:**
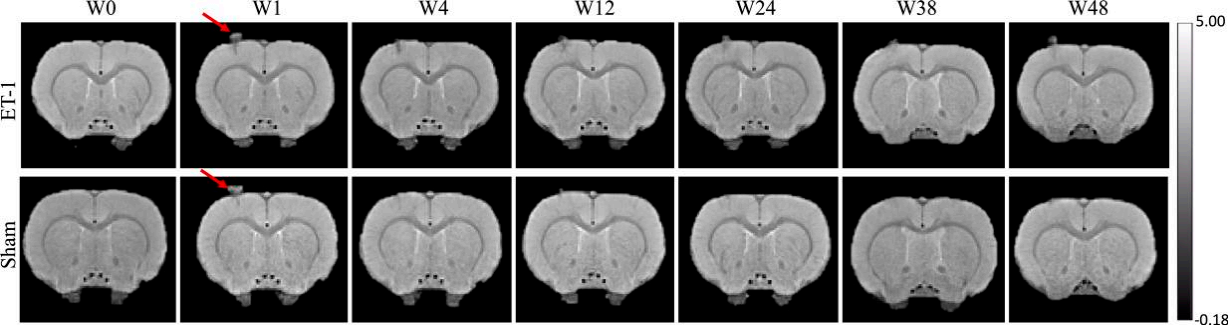
T_2_*w template images. Exemplar template images are shown for stroke and sham rats at baseline and Weeks 1, 4, 12, 24, 36 and 48. Endothelin-1 (ET-1) and saline injection sites are visible in the right motor cortex (arrows). The images are displayed in radiological convention (left: ipsilateral side)

In all stroke rats, ET-1 injections induced focal infarcts in the right motor cortex visible on T_2_*w images (Fig. 1).

**Figure 2:**
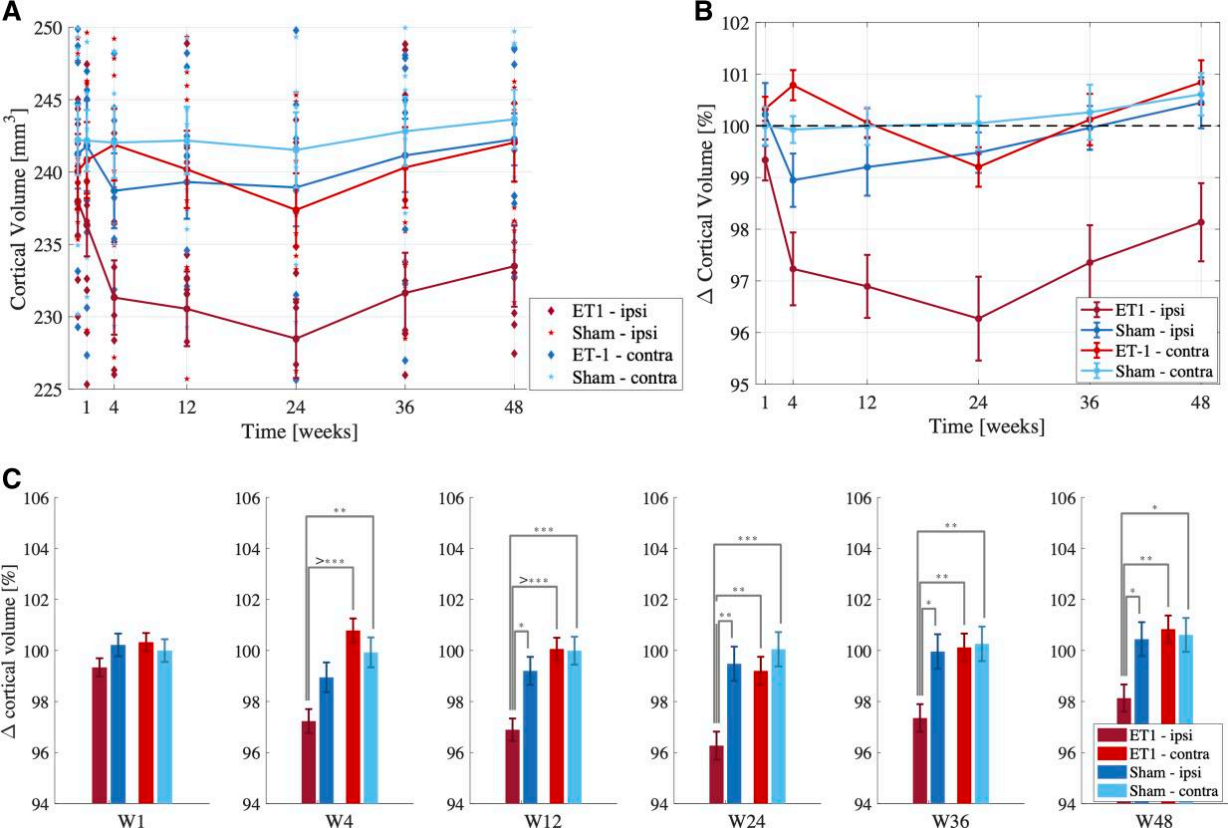
**Cortical volume trajectories (mean ± SE).** The cortical volume trajectories are shown from baseline to 8 weeks in stroke (ipsilesional: maroon, contralesional: red) and sham (ipsilateral: blue, contralateral: cyan) rats. (**A**) Cortical volume (mm^3^) and (**B**) percent volume change compared with baseline (mean ± SE). (**C**) *Post hoc* pairwise comparisons at each timepoint [four groups: stroke ipsilesional (*n* = 14 animals), stroke contralesional (*n* = 14 animals), sham ipsilateral (*n* = 9 animals), sham contralateral (*n* = 9 animals)]. At Week 24, *n* = 12 in the stroke group and *n* = 8 in the sham group. Ipsilesional cortex is smaller in stroke rats 12–48 weeks post-stroke compared with ipsilateral sham cortex and contralateral cortices in both groups (*P < 0.01, **P < 0.001, ***P < 0.0001).

Cortical atrophy was observed ipsilesionally in stroke rats over time (Fig. 2A). After an initial 2.7% reduction at 4 weeks, ipsilesional volume loss peaked at 24 weeks (4% compared with baseline), followed by a recovering trend at Weeks 36 and 48 (Fig. 2B).

Linear mixed-effect regression modelling showed significant three factor interactions (group/side/time) from Weeks 4 to 48 (*t*_(282)_ = 2.85, 2.63, 2.42, 2.74, 2.81, *P* = 4.8e-3, 9.0e-3, 1.6e-2, 6.6e-3, 5.4e-3), suggesting differential bilateral evolution of cortical volume trajectory in stroke and sham rats over time. Follow-up analyses showed significant ipsilesional volume decreases at each timepoint compared with baseline volume, with sustained ipsilesional cortical atrophy in stroke rats over time. *Post hoc* cross-sectional comparisons at each timepoint separately using normalized cortical volumes showed significant group/side interactions at weeks W4 (*F*_(45)_ = 5.87, *P* = 0.02), W12 (*F*_(45)_ = 5.72, *P* = 0.02) and W48 (*F*_(45)_ = 4.44, *P* = 0.04). At Weeks W24 and W36, there were significant main effects of group (*F*_(39)_ = 10.94, *F*_(45)_ = 4.98) and side (*F*_(39)_ = 8.15, *F*_(45)_ = 6.24). Smaller ipsilesional cortices were observed in stroke rats compared with ipsilateral sham and contralateral cortices in stroke and sham rats.

### Hippocampal and thalamic volume trajectories

**Figure 3:**
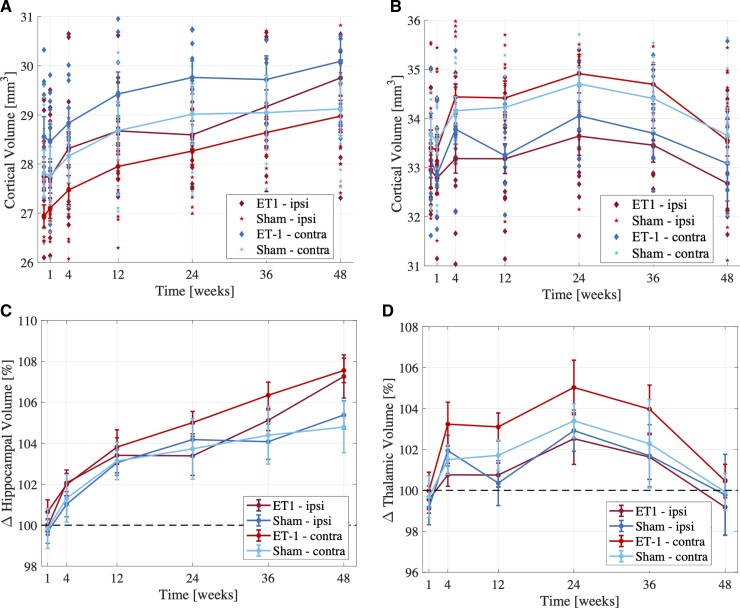
**Trajectories of regional brain volumes.** Regional brain volume changes (mean ± SE) from baseline to 48 weeks post-stroke onset in (**A**) hippocampus and (**B**) thalamus. Percent volume change compared with baseline volume (mean ± SE) in (**C**) hippocampus and (**D**) thalamus. Linear mixed effects modelling showed significantly larger hippocampi in both stroke and sham rats at Weeks 12–48 compared with baseline volume. At Week 48, stroke rats had marginally larger hippocampi than shams (∼2% difference, *P* = 0.019). Larger thalamic volumes were observed in stroke and sham rats up to 24 weeks, with peak volume increase of ∼4% compared with baseline (*P* = 2.53e-4). After Week 24, decreasing thalamic volumes were observed in all animals, reaching baseline at 48 weeks. All statistical analyses compared four experimental groups: stroke ipsilesional (*n* = 14 animals), stroke contralesional (*n* = 14 animals), sham ipsilateral (*n* = 9 animals), sham contralateral (*n* = 9 animals).

Linear mixed modelling showed significantly larger hippocampi in both stroke and sham rats at Weeks 12–48 (*t*_(282)_ = 5.05, 6.68, 8.13, 9.97, *P* < 0.0001) compared with BLV (Fig. 3A and B), potentially revealing age-related hippocampal changes. At 48 weeks, stroke rats had marginally larger hippocampi than shams (∼2% difference, *P* = 0.019).

Liner mixed modelling of thalamic volumes showed no significant main group or side effect or interactions. Significant main effects for time were present from Weeks 4 to 36. Larger thalamic volumes were observed in stroke and sham rats up to 24 weeks, with peak volume increase of ∼4% compared with baseline (*t*_(282)_ = 3.72, *P* = 2.53e-4). After 24 weeks, decreasing thalamic volumes were observed in all animals, reaching baseline at 48 weeks.

### Tensor-based morphometry

In stroke rats, TBM analyses identified ipsilesional regions of cortical atrophy at 4, 24 and 36 weeks after stroke onset at a statistical significance level of *P* ≤ 0.1 after family-wise error correction (Supplementary Fig. 1), however these foci of cortical atrophy did not survive at *P* ≤ 0.05.

### Fractional anisotropy changes after ischaemic injury

**Figure 4:**
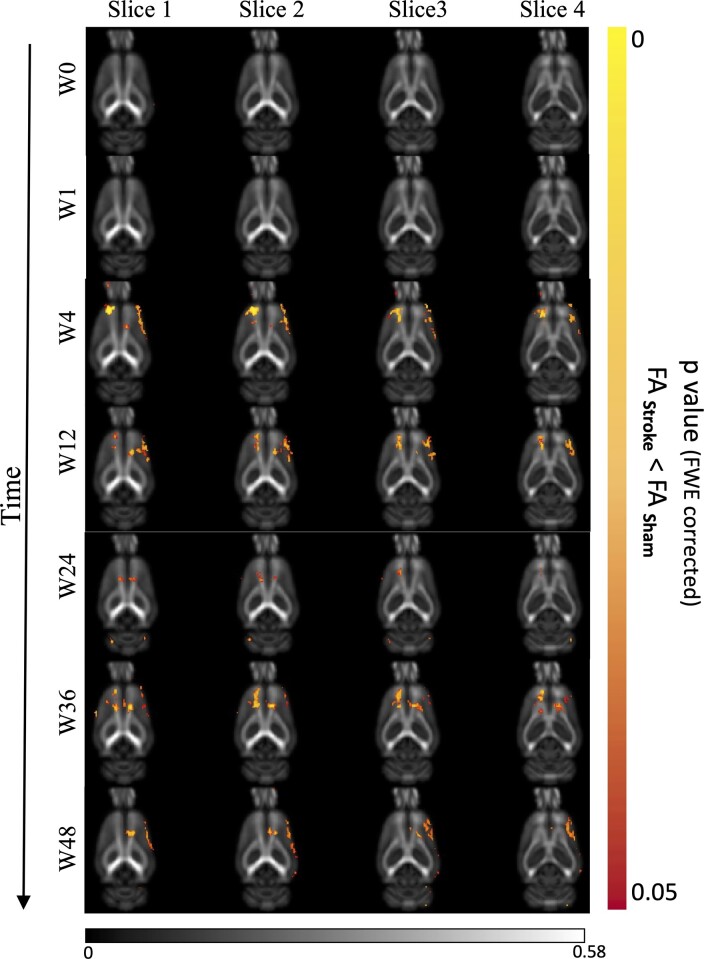
**Changes in white matter post-stroke.** Fractional anisotropy (FA) changes from baseline to 48 weeks post-stroke (left: contralateral side). Voxel-wise unpaired *t*-tests (14 stroke animals and 9 sham animals) revealed significant FA reduction in both hemispheres, starting from 4 weeks and persisting up to 48 weeks after stroke onset. Exemplar FA study template axial slices overlaid with *P*-value map (TFCE, FWE-corrected). Right: ipsilateral size.

In stroke animals, we found significantly reduced FA in motor cortex and cingulum bundle regions of ischaemic brains both ipsi- and contralesionally (*P* < 0.05) from 4 to 36 weeks post-stroke suggest long-term compromised WM integrity in distant regions (Fig. 4, Supplementary Fig. 2). At 48 weeks, only an ipsilesional decrease in FA was observed. Regions of reduced FA were within motor networks (Supplementary Fig. 3). No significant changes were observed in MD/AD/RD.

## Discussion

These results show that a small cortical stroke in rats can lead to persistent changes in brain integrity both in grey and WM, with diffuse atrophy of the ipsilesional cortex and alterations in WM integrity (reduced FA) in the cingulum bundle up to 48 weeks post-stroke.

Following ET-1 injection in the motor cortex of rat, we found a persistent reduction in overall cortical volume in the ipsilateral hemisphere of stroked rats compared with their initial cortical volume. This reduction was observed from 4 weeks post-stroke and persisted until 48 weeks, with a peak at 24 weeks corresponding to a reduction of 4% of the overall ipsilateral cortex volume. This finding was specific to the stroke group. This result is consistent with previous results describing long-term pathohistological changes following mild ischaemic injury.^28^

Progressive remote atrophy is a hallmark of SND. In an effort to characterize the SND in our model of minor stroke, we used T_2_*w images to measure the volume of the hippocampus and thalamus. Hippocampal and thalamic volume measured on T_2_* images were not different in the stroke rats, except for a 2% increase of hippocampal volume in stroke rats compared with control at 48 weeks; however, we suspect this small difference to be due to variability in brain volume and small stroke volumes in this endothelin-1 model. This is in contrast to the more severe stroke studies.^15,16^ We therefore suggest that these remote changes are severity-dependent and in our model of mild stroke the SND is limited to the cortex. Interestingly, we found an increase over time for overall hippocampal volume for both groups, corresponding to a ∼6% increase at 48 weeks, equivalent to the rats being 5.5- to 17.5-months old. This pattern has previously been described in rodent studies from our group^43^ and others,^44,45^ likely representing the normal maturation of healthy brains.

An interesting finding is that of atrophy peaking at 24 weeks post-stroke then reversing. An obvious explanation is that this may represent a remodelling effect. Cortical thickness decreases (e.g. Lotan)^46^ and increases (Brodtmann)^47^ have been observed following focal stroke. In this latter small pilot study of people 3 months after ischaemic stroke, we noted that cortical thickness increases in contralesional paracentral, superior frontal and insular region were in areas known to be activated in functional MRI studies of motor recovery. Alternatively, it may be part of the normal aging process in rodents, which we have incidentally captured by performing longitudinal imaging over such a long time-frame in our animals. In a separate mouse MCAo study performed by our group,^43^ we found a progressive increase in hippocampal volume bilaterally from 10 to 30 weeks of age, suggesting that hippocampi continue to increase in size with increased age. We found no change in hippocampal volume from 30 to 54 weeks of age, suggesting that hippocampal atrophy does not occur in normal aging in the mouse, at least up to the 54 weeks of age at which we sacrificed our mice.

One of the most interesting findings from this study was the FA changes observed in the cortex and cingulum bundle of both hemispheres from 4 to 48 weeks post-stroke. WM changes have been mostly overlooked as a mechanism for post-stroke neurodegeneration and cognitive decline, with existing studies principally focusing on grey matter changes. We have previously reported pervasive WM degeneration at 3 months after stroke.^12^ Visser *et al.*^48^ also showed an increase in FA in contralesional primary motor cortex of stroke patients 3 months post-stroke.^48^ This is consistent with our finding, which showed a localized WM integrity change with FA changes in the cortex, which coincided with the prolonged atrophy seen on the T_2_* images. This was further seen by the TBM analysis, which revealed that the cortical volume loss is more focal in the motor cortex at 4, 24 and 36 weeks, whereas at Weeks 1, 12 and 48 the loss is more dispersed across the whole cortex, suggesting varying temporal courses of cortical atrophy. Similar FA change in the corticospinal tract has been seen in humans as early as 30 days post-stroke and was associated with poor motor performance.^49,50^

The changes in the cingulum bundle were however more surprising. The cingulum contains prominent medial and dorsal prefrontal connection and has been found to have a role in executive function, decision-making and emotion processing.^51,52^ Furthermore, cingulate impairment is implicated in a wide range of diseases depending on the lesion site, including Alzheimer disease, schizophrenia, depression, post-traumatic stress disorder, obsessive compulsive disorder and autism.^53–55^ Diffusion MRI studies in humans have shown that cingulum lesions produce mild cognitive deficits, with one study suggesting that the cingulum microstructure can predict cognitive control/flexibility in older age and in mild cognitive impairment^56,57^ and structural changes in MD in dorsal cingulum bundle were associated with longer reaction time.^58^ A recent study also showed a direct correlation between the reduction in FA in the WM tracts and poor patient recovery and suggested that FA changes in the corpus callosum in the first 3 months post-stroke could predict the patient’s recovery.^59^ Elevated ipsilesional versus contralesional MD of the cingulum bundle has been demonstrated in human ischaemic stroke survivors.^3^ We have previously shown post-stroke atrophy in the cingulum in a group of ischaemic stroke patients at 3 months^12^ and have posited that this WM atrophy may represent disconnection syndromes underlying post-stroke cognitive impairment.^13,60–62^ However, to our knowledge, this is the first time that cingulum bundle disruptions have been linked to mild stroke, either in humans or in a rodent model.

The findings from this study should be interpreted in the context of several limitations. The TBM provides a measure of voxel-wise regional volumetric changes with respect to a reference template, such as the MDT constructed from the baseline data in this study to reduce longitudinal and procedural bias in the analyses.^63^ Several other templates can be used as a reference, such as a study template from all groups and timepoints included in the study, and the choice of template is an important factor to consider in the interpretation of the log-Jacobian metric. Furthermore, changes in the fractional anisotropy measures can be driven by a combination of axonal loss, demyelination, or morphometric alterations in WM fibre bundles, and it is not possible to determine the specific biological mechanisms underpinning these changes without additional histological information or the use of more advanced diffusion models.^64^ Given the longitudinal design of the study, it is not possible to conduct histology at all timepoints included in the study. However, our group has recently published a separate companion study with similar ET-1-induced stroke lesion (Ermine *et al.*).^31^ In this study we described results from the histological assessment at different time points (1, 4, 12, 24, 36 and 48 weeks) and showed a minimal neuronal loss in shams at the injection site at 1 week but resolved by 4 weeks. This is further validated by the emergence of group differences four week after surgery in the ipsilesional cortical volume, TBM and FA metrics in the current study.

## Conclusion

Long-term cortical atrophy and WM changes were observed in an endothelin-1 model of ischaemic stroke and contralateral WM changes in regions connected to the initial infarction site provide evidence of SND. This study offers further insight into the damage from a mild stroke and provides further information on the mechanism leading to the cognitive decline and dementia post-stroke. The role of WM integrity is becoming increasingly recognized for a range of neurodegenerative disorders including post-stroke cognitive impairment.

## Supplementary Material

fcac185_Supplementary_Data

## Abbreviations

AD =: axial diffusivity
DTI =: Diffusion Tensor Image
DWI =: diffusion-weighted imaging
ET-1 =: endothelin-1
FA =: fractional anisotropy
FOD =: fibre orientation distribution
MCAo =: middle cerebral artery occlusion
MD =: mean diffusivity
MDT =: minimum deformation template
PCA =: posterior cerebral artery
RD =: radial diffusivity
ROIs =: regions-of-interest
SND =: secondary neurodegeneration
STAIR =: Stroke Therapy Academic Industry Roundtable
T_2_*w =: T_2_*-weighted
TIA =: transient ischaemic attack
TBM =: tensor-based morphometry
WM =: white matter
