# Supplementary material for: Long-term structural brain changes in adult rats after mild ischaemic stroke
Source: Brain Commun. 2022 Jul 22;4(4):fcac185. doi: 10.1093/braincomms/fcac185 (PMC9309495; doi:10.1093/braincomms/fcac185)
Supplement: fcac185_Supplementary_Data [file fcac185_supplementary_data.docx]

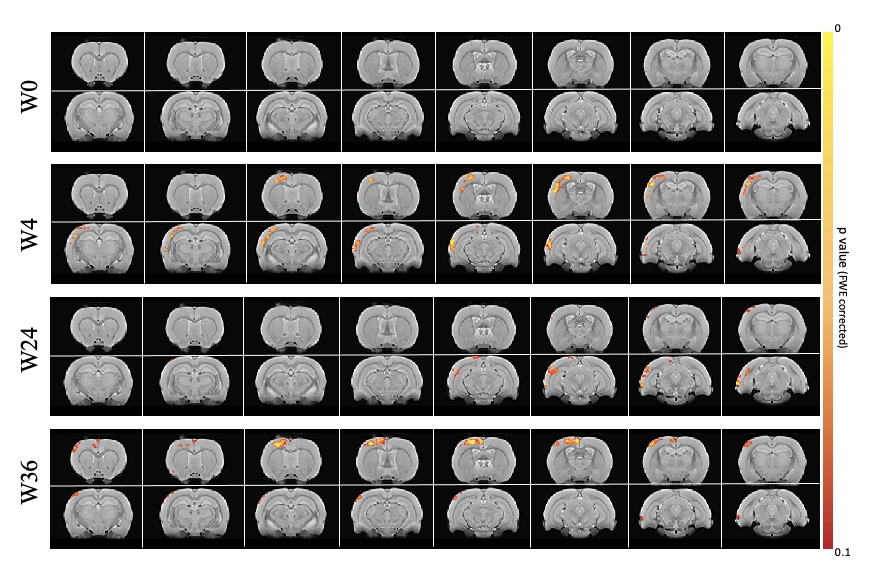


**Supplementary Figure 1:**

Voxelwise cortical volumes atrophy identified by tensor-based morphometry at baseline and weeks 4, 24 and 36 displayed on the study template after family-wise error correction at 10% significance level ($\alpha=0.1) (Left: ipsilateral side, displayed in radiological convention)$. The displayed foci of cortical atrophy did not survive at 5% significance level. No focal cortical atrophy was identified at weeks 1,12 and 48 at any significance level.


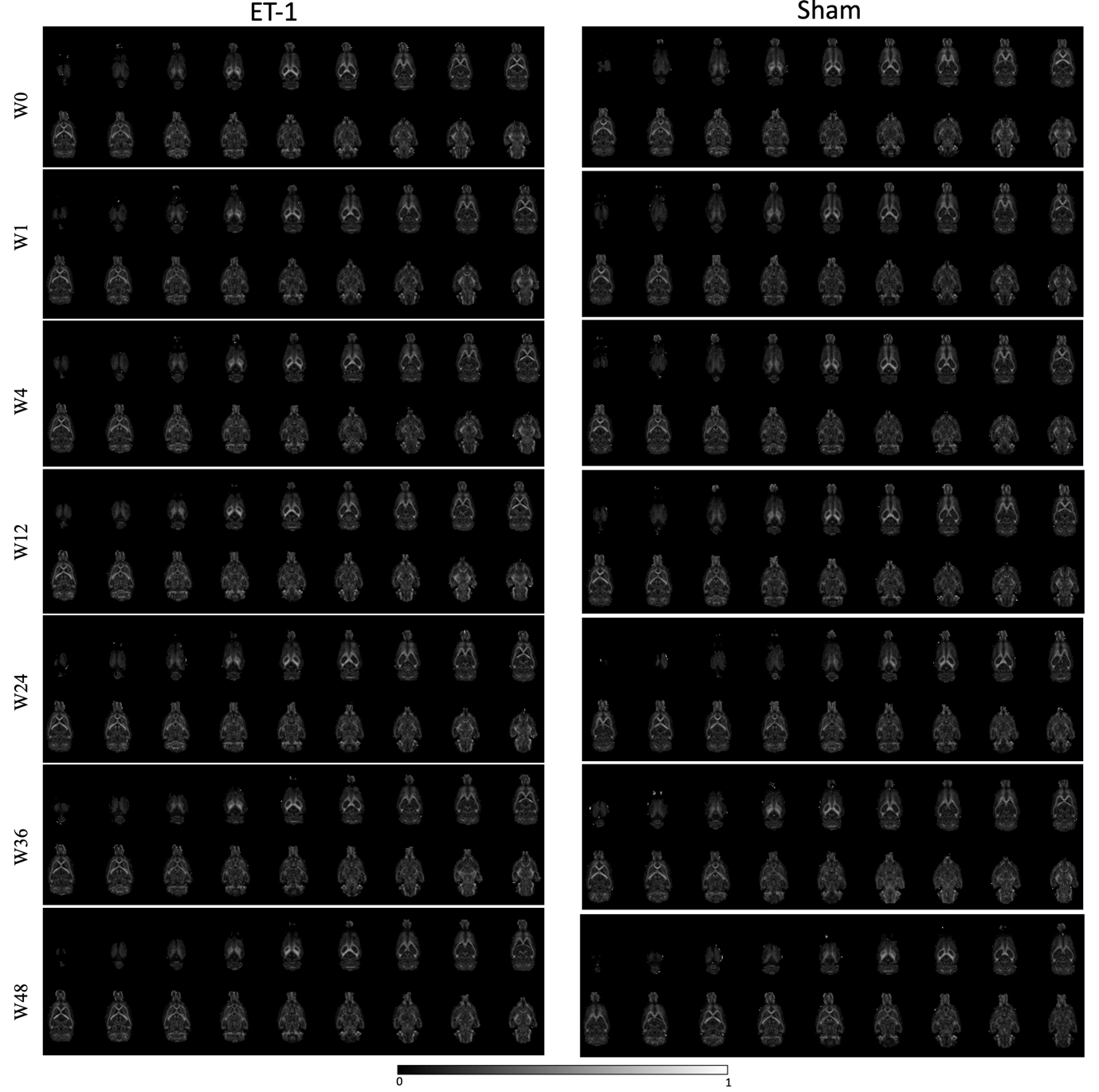


**Supplementary Figure 2:**

Exemplar FA maps from an ET-1 and a sham rat at all timepoints before registration and template construction steps. Right: ipsilateral side.

**Supplementary Figure 3:**

Connectivity of right motor cortex (ET-1 injection site) to remote brain regions. Exemplar FA study template axial slices overlaid with A) regions of significant FA decrease at 36 weeks post-stroke and B) tractograms demonstrating connectivity between affected regions and right motor cortex (displayed on the right side).
